# Molecular Epidemiology of *Mycobacterium tuberculosis* strains isolated from pulmonary tuberculosis patients in south Ethiopia

**DOI:** 10.3855/jidc.14742

**Published:** 2021-09-30

**Authors:** Yared Merid, Elena Hailu, Getnet Habtamu, Melaku Tilahun, Markos Abebe, Mesay Hailu, Tsegaye Hailu, Daniel G Datiko, Yimtubezinash Woldeamanuel, Abraham Aseffa

**Affiliations:** 1 College of Medicine and Health Sciences, Hawassa University, Hawassa, Ethiopia; 2 College of Health Sciences, Addis Ababa University, Addis Ababa, Ethiopia; 3 Armauer Hansen Research Institute, Addis Ababa, Ethiopia; 4 Management Sciences for Health, Addis Ababa, Ethiopia

**Keywords:** Molecular epidemiology, Tuberculosis, drug susceptibility testing, geospatial cluster, Ethiopia

## Abstract

**Introduction:**

Understanding the epidemiology of tuberculosis is limited by lack of genotyping data. We sought to characterize the drug susceptibility testing patterns and genetic diversity of *M. tuberculosis* isolates in southern Ethiopia.

**Methodology:**

A cross-sectional study was conducted among newly diagnosed sputum smear positive patients with tuberculosis visiting nine health facilities in southern Ethiopia from June 2015 to May 2016. Three consecutive sputum samples (spot-morning-spot) per patient were examined using acid-fast bacilli smear microscopy with all smear positive specimens having acid-fast bacilli cultures performed. *M. tuberculosis* isolates had drug susceptibility testing performed using indirect proportion method and were genotyped with RD9 deletion analysis and spoligotyping. Mapping of strain was made using geographic information system.

**Results:**

Among 250 newly diagnosed patients with tuberculosis, 4% were HIV co-infected. All 230 isolates tested were *M. tuberculosis* strains belonging to three lineages: Euro-American, 187 (81%), East-African-Indian, 31 (14%), and Lineage 7 (Ethiopian lineage), 8 (4%); categorized into 63 different spoligotype patterns, of which 85% fell into 28 clusters. *M. tuberculosis* strains were clustered by geographic localities. The dominant spoligotypes were SIT149 (21%) and SIT53 (19%). Drug susceptibility testing found that 14% of isolates tested were resistant to ≥ 1 first line anti- tuberculosis drugs and 11% to INH. SIT 149 was dominant among drug resistant isolates.

**Conclusions:**

The study revealed several clusters and drug resistant strains of *M. tuberculosis* in the study area, suggesting recent transmission including of drug resistant tuberculosis. Wider monitoring of drug susceptibility testing and geospatial analysis of transmission trends is required to control tuberculosis in southern Ethiopia.

## Introduction

Tuberculosis (TB) remains a major and urgent global public health problem, especially in low-income countries where the burden of disease is high. Globally, there were 10 million new cases and 1.2 million deaths among persons with TB in 2019. Drug-resistant TB is a major public health concern in many countries and continues to be a public health threat [1]. Ethiopia has a long history of TB [2] and is still hugely affected by the disease and it is one of fourteen countries appearing in all three WHO high burden country lists for TB, TB/HIV and MDR-TB [1]. According to the national anti-tuberculosis drug resistance survey in 2014, the prevalence of MDR-TB among new and previously treated TB cases was 2.3% and 17.8% respectively [3]. Additionally, data from different parts of the country show that drug-resistant TB is a major public health concern that demands attention [4,5].

Interruption of the transmission of *M. tuberculosis* is one of the primary goals of tuberculosis control programs. Tracking specific strains of *M.tuberculosis* circulating in the community informs public health authorities on patterns of spread and potential areas for action to curb the spread of TB in communities [6]. Spoligotyping is a PCR-based method commonly used to characterize *M. tuberculosis* strains circulating in a community [7] and strain differentiation is based on the polymorphism in the direct repeat (DR) locus, which is a distinct chromosomal region in *M. tuberculosis* genome [8]. Although the current trend is recommending the use of a combination of typing methods to provide a higher resolution and better characterize than spoligotype alone, the use of spoligotype would still provide useful information relative to high burden settings with limited recourses. It has been extensively used for simultaneous detection and typing of *M. tuberculosis* [7].

The lack of comprehensive molecular epidemiological data from most countries in Africa, such as Ethiopia, has limited the understanding of TB disease dynamics. While several molecular epidemiological studies have been conducted to describe the diversity and drug susceptibility profile of *M. tuberculosis* strains in various geographical areas of Ethiopia [9–11], data from the south, where population density is relatively higher, is limited. Moreover, there is no data on the spatial distribution of *M. tuberculosis* clustered strains in the country. In order to have a clear understanding of the ongoing transmission dynamics of *M. tuberculosis* in a community, GIS mapping supported cluster position studies are recommended [12]. The goal of our study was to characterize the drug susceptibility patterns and genetic diversity of *M. tuberculosis* isolates circulating in southern Ethiopia.

## Methodology

### Study design and area

**Figure 1.**
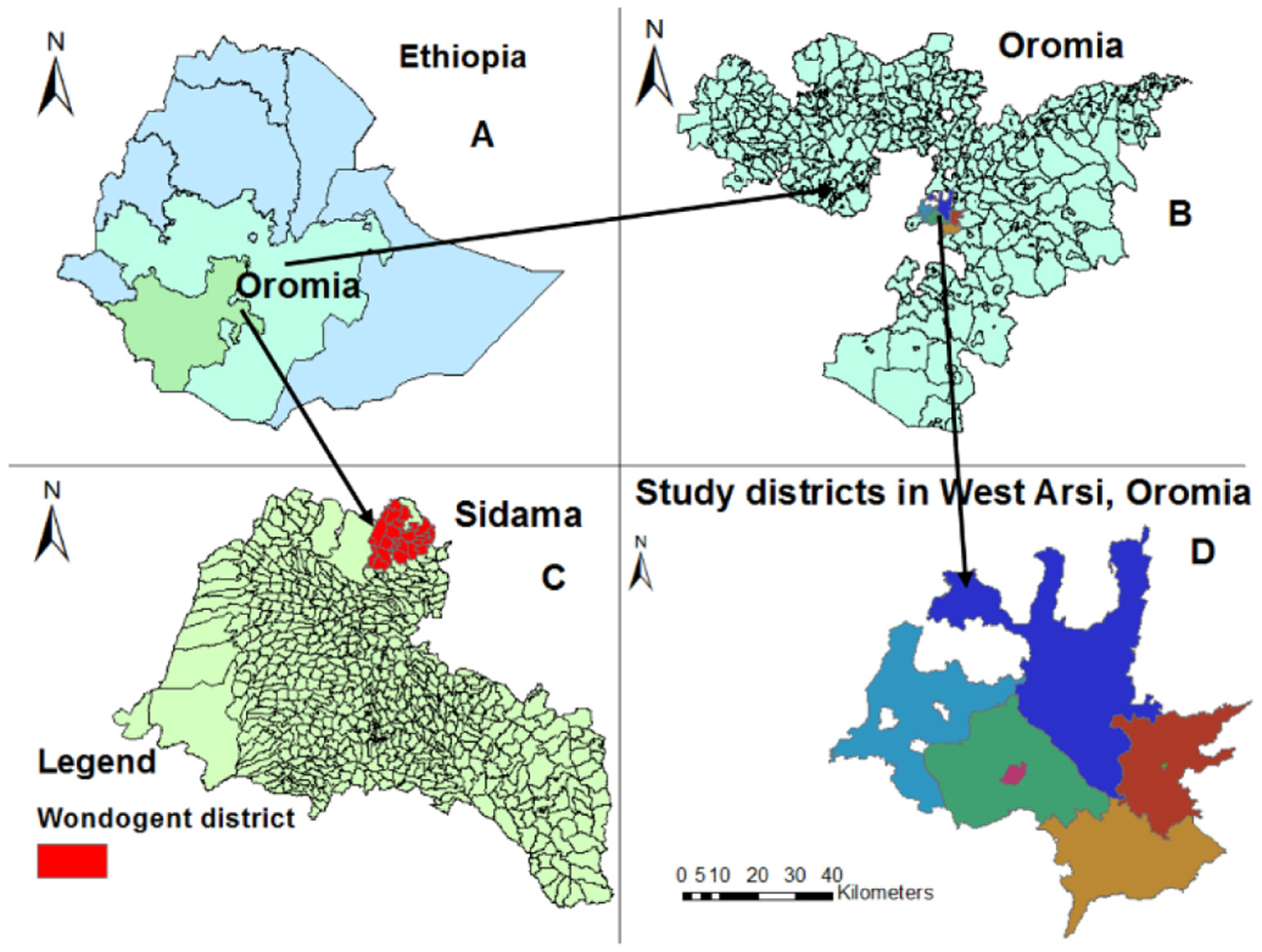
Map of the study area (East Arisi Zone districts, Oromia and Wondo Genet of Sidama Regional State) A: Map of Ethiopia; B: Map of Oromia Regional State; C: Map of districts of Oromia Regional state and Wondogenet district from Sidama Regional State; D: Map of districts involved in the study.

This cross-sectional study was conducted at nine health facilities (two hospitals and seven health centers) in and around Shashemene area, in West Arisi Zone of Oromia Region, Ethiopia. Shashemene is a major urban center and commercial town, located 240 km south of the capital Addis Ababa. The estimated population of the study area was 3 million people. All nine health facilities included in the study area provided services for the diagnosis and treatment of TB through DOTS clinics. Patients enrolled at the nine health facilities from the West Arsi Zone and adjoining kebele of Wondogenet were mapped and fell into seven districts (Woredas) (Figure 1).

### Study population and variables

Among the persons suspected to have TB who were investigated at any one of the nine health facilities during the study period, all newly diagnosed sputum smear positive pulmonary TB patients who provided written informed consent for study participation were enrolled. Assent for the children and consent by parents or guardians for those under 18 yrs of age were also obtained. The study was conducted from June 2015 through May 2016. Three consecutive sputum samples (spot-morning-spot) were collected from each TB suspect. Socio-demographic and clinical information was obtained for all study participants by a TB clinic nurse at the respective health facilities using a pre-tested standard questionnaire. Data on HIV status was retrieved from health facility records.

### Definition

Newly diagnosed patients with pulmonary TB refer to patients who had never been previously treated for TB or have only taken anti-TB drugs for less than 1 month. *M. tuberculosis* isolates (two or more) that share the same genotype based on spoligotyping were considered clustered.

### Laboratory methods

#### Specimen collection

Sputum samples were collected at each respective health facilities using sterile sputum cups; sputum smears were prepared at the same day as the sputum collection and examined by an onsite health facility laboratory technologist. The remaining portion of the three sputum samples from AFB smear positive patients were pooled individually into 50 mL sterile screw capped universal test tubes and stored at the diagnostic centers at −20 °C for a maximum of one week until transported on cold chain to the Armauer Hansen Research Institute (AHRI) in Addis Ababa, Ethiopia for mycobacterial culture, RD9 deletion analysis and spoligotyping.

#### Mycobacterial culture

The pooled sputum samples were processed at AHRI within one day based on standard procedures as previously described [13]. In brief, the sputum samples were digested and decontaminated using Petroff’s method and the processed sample was inoculated into three tubes containing egg-based Löwenstein Jensen (LJ) media (two with glycerol and one with pyruvate). The inoculated media were incubated at 37 °C for at least 8 weeks, with weekly observation for the presence of mycobacterial colonies. Cultures with no growth after the eighth week were considered negative. Mycobacterial growth was confirmed by typical colony morphology and AFB staining.

#### Drug susceptibility test (DST)

The conventional indirect proportion method was employed to perform DST using 7H10 medium on 24-well tissue culture plates using a standard protocol [14]. In brief, four first line anti-TB drugs (isoniazid, rifampicin, ethambutol and streptomycin) were mixed with the agar media at recommended concentration and dispensed into 9 wells of the 24 well tissue culture plates and two wells were dispensed with drug free media. The agar plate was sealed with parafilm and incubated in an inverted position at 35 °C. The plates were checked on day 6, 12 and 19 for evidence of growth. Resistance was expressed as the percentage of colonies that grew on critical concentrations of the drugs, i.e. 0.2 μg/mL for isoniazid (INH), 1μg/mL for rifampicin (RPM), 5μg/mL for ethambutol (EMB) and 2μg/mL for streptomycin (STM). The interpretation of resistance was based on the standard criteria for resistance, i.e. 1% for all drugs [15].

#### DNA extraction

Mycobacterial genomic DNA was extracted by heating the isolates at 80 °C in a sonicator water bath for an hour [16].

#### RD9 deletion analysis

Region of difference 9 (RD9) deletion analyses was performed on heat-killed cells to confirm the presence or absence of RD9 for species identification of *M. tuberculosis* from the other members of MTBC as previously described [17]. It uses three primers (RD9flankF, RD9intR and RD9flankR) for PCR reaction. PCR amplification of the mixtures was done using a Thermal Cycler PCR machine. The PCR amplification product was run by electrophoresis in 1.5% agarose gel in 1× Trisacetate- ethylene diamine tetraacetic acid running buffer at 110volt for 35 minutes. Ethidium bromide at a ratio of 1:10, 100 base pair (bp) DNA ladder, and orange 6× loading dye was used in gel electrophoresis and the gel was visualized. The results were interpreted as *M. tuberculosi*s when a band of 396 bp was observed (RD-9 positive). Detection of a band size of 575 bp was considered to be positive for the other members of *M*. *tuberculosis* complex species (*M. bovis* or *M. africanum).* DNA from *M. bovis* BCG and *M. tuberculosis* H37Rv were used as positive controls, whereas autoclaved ultrapure water was used as a negative control.

#### Spoligotyping

Isolates that were positive for *M. tuberculosis* by RD9 PCR were further characterized by spoligotyping following the procedure described earlier [7]. In brief, the direct repeat (DR) region of the isolate was amplified by PCR using oligonucleotide primers (DRa and DRb) derived from the DR sequence. Individual spoligotyping patterns were compared with the recent International Spoligotyping Database (SITVITWEB). Spoligotyping International Types (SIT) and sub-lineages (clades) were assigned according to signatures provided in SITVITWEB data base [18]. An isolate was defined as a shared type if the same spoligotype was found in the database. If no matching spoligotype was found in the database, the isolate was defined as orphan (new).

#### Data management

Data were double entered into an online REDCap database [19] and analyzed using STATA v1 (StataCorp, College Station, TX, USA).

#### Spatial analysis

Mapping of TB lineage and strain clusters was made using GIS. First, the proportion of different types of TB lineages was computed (stratified) by district to look for variations in geographic distribution of lineages. Second, clustered strains were mapped by geographic locations. ArcGIS software (10.2) was used for mapping the geographic distribution of lineages and clustered strains by district. The shape file of study districts were obtained from Central Statistics Agency of Ethiopia (CSA). A geographic projection of the World Geodetic System (WGS), Universal Transverse Mercator (UTM) Zone 37 N was used for analysis. The data of attributes (number and proportion of TB cases, lineages and TB strains) were geo-linked for each district with the geographic data (shape file) using feature identification.

### Ethical consideration

The study was approved by the Institutional Review Boards of Addis Ababa University and the Armauer Hansen Research Institute (AHRI) as well as the Ethiopian National Ethics Review committee. Study permission was also obtained from the Oromia Regional Health Bureau, Western Arisi zone Health Department, Southern Regional Health Bureau and Sidama Regional Health Bureau.

## Results

### Socio-demographic characteristics

Among 250 newly diagnosed sputum smear positive TB patients enrolled, 145 (58%) were male and 143 (57%) from urban areas (Table 1). The median age was 25 years (interquartile range [IQR] 20–30). One hundred twenty-nine (52%) were married, 195 (78%) had an educational level of at least primary school. Farmers, students and house wives altogether accounted for 70% (174) of the study participants. TB-HIV co-infection was present in 10 (4%).

### Genetic diversity of strains

**Figure 2.**
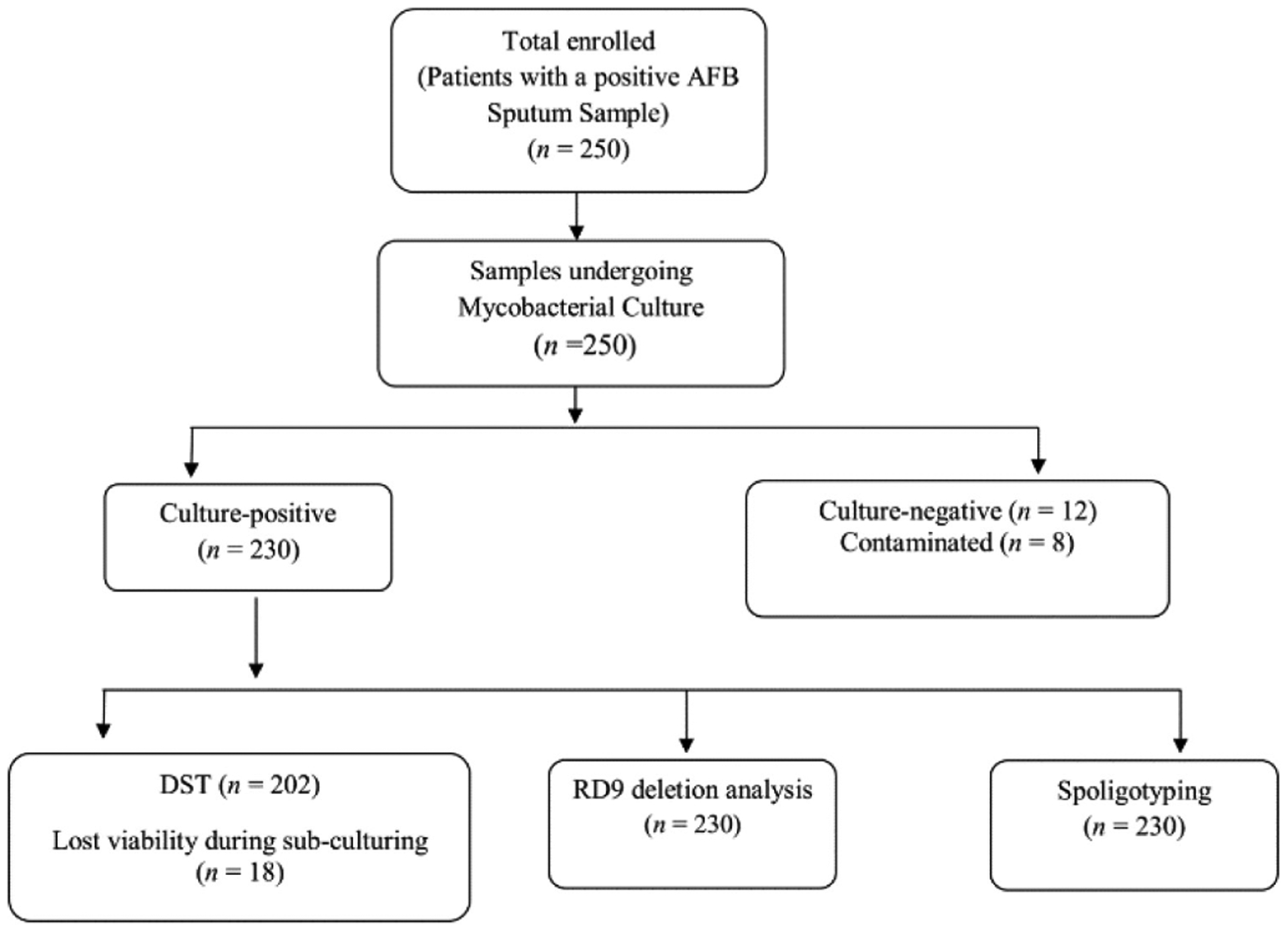
Study Diagram. DST: drug susceptibility testing; RD9: region of difference-9.

All 250 AFB positive sputum samples had mycobacterial culture performed; 230 (92.0%) were positive, 8 (3.2%) were contaminated and 12 (4.8%) failed to grow on culture (Figure 2). The 230 isolates were all identified as *M. tuberculosis* by RD9 deletion analysis. Spoligotyping analysis identified 63 spoligotype patterns, of which 35 (56.0%) were already known in the international data base and 28 (44.0%) were new patterns (orphans).

**Figure 3.**
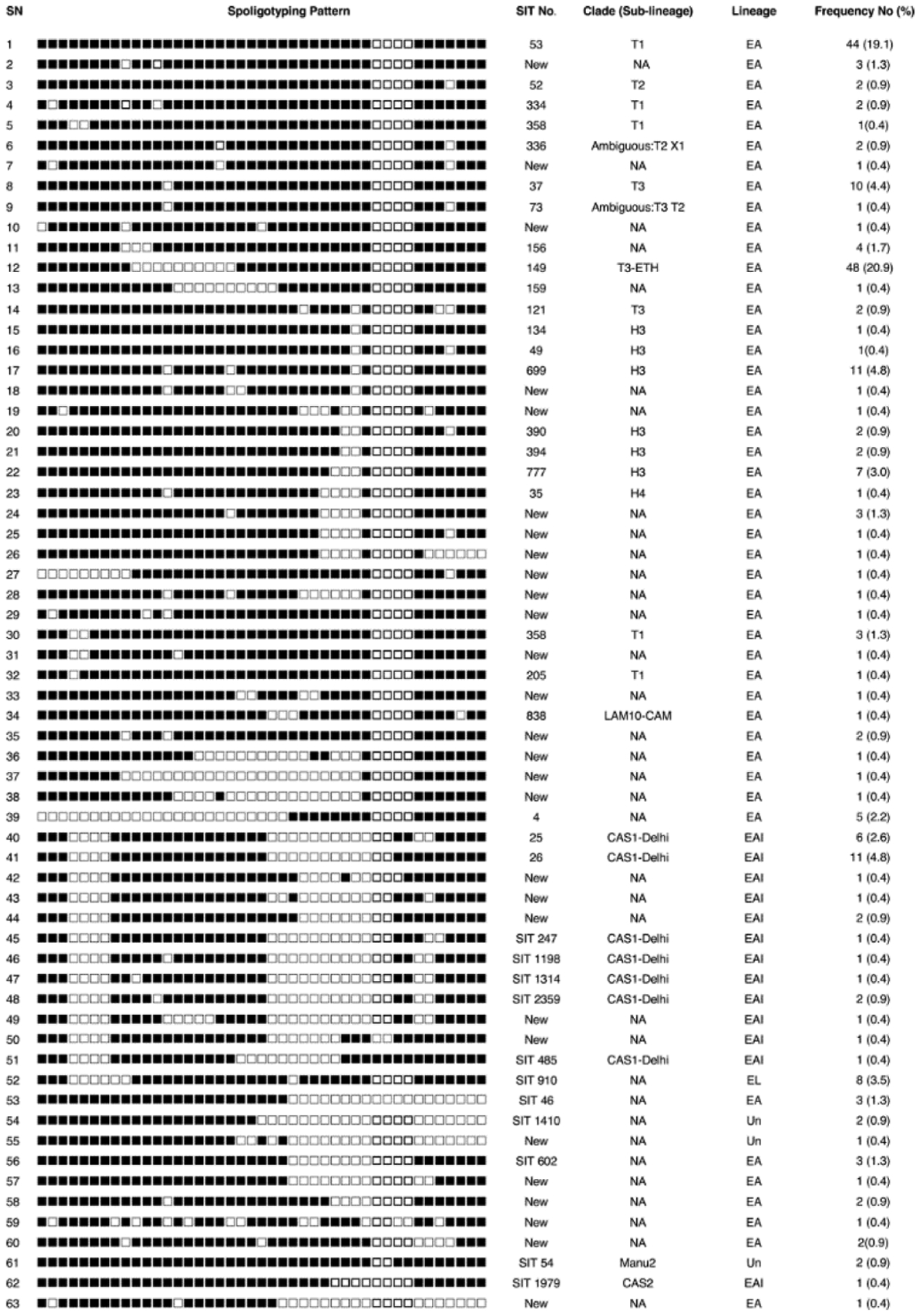
Spoligotype pattern of *M. tuberculosis* strains isolated from pulmonary tuberculosis patients in southern Ethiopia. The black squares represent positive hybridization signals and white squares represent a lack of hybridization. EA: Euro-American; EAI: East-African-Indian; EL: Ethiopian lineage (L7); SIT: Spoligotype international type; NA: Not assigned; Un: unknown.

The lineage distribution showed that 187 (81%) isolates belonged to the Euro-American lineage (L4), 31 (14%) to East-African-Indian (L3) and 8 (4%) to Lineage 7 (Ethiopian lineage). Three strain types could not be assigned to any of the lineages. The predominant clade (sub-lineage) was T1 (51, 22%), followed by T3-ETH (48, 21%), H3 and CAS1-Delhi (23, 10%) each (Figure 3).

The most dominant shared types were SIT149 (48, 21%) and SIT53 (44, 19%) while (37, 16%) were orphan strains (of different spoligotype patterns). One hundred ninety-five (85%) of the isolates were clustered into 28 spoligotype patterns and the remaining (36, 16%) strains fell into single spoligotypes. Cluster size varied from 2 to 48 strains per cluster. Of the clustered 195 strains, 181(93%) were already registered in the international database and the other 14 (7%) were orphans. Sixty-two (27%) of the strains were not assigned for clade in the SITVITWEB database [18].

#### Mapping of TB lineage and strain clusters

**Figure 4.**
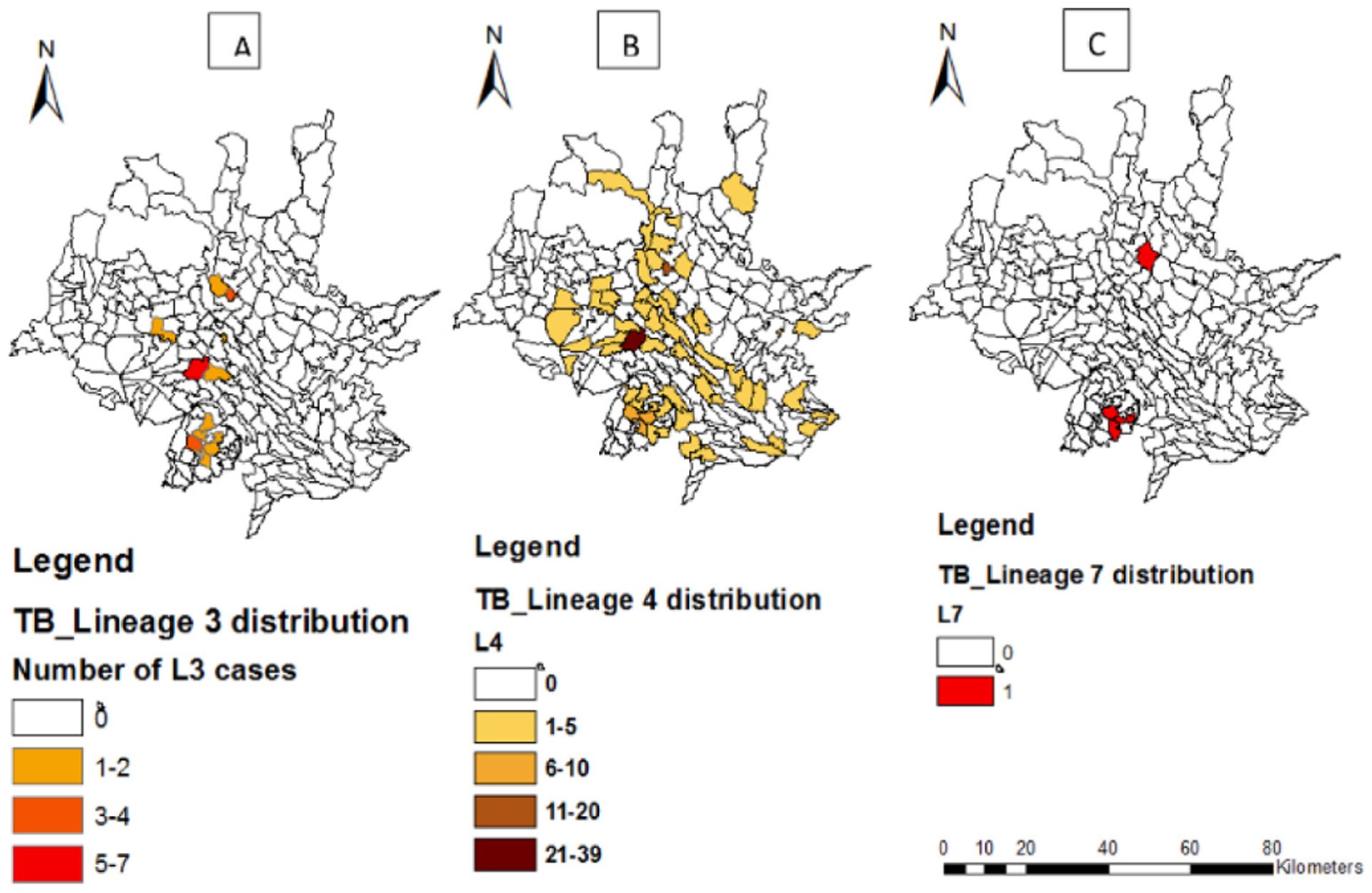
Spatial distribution of TB lineages; West Arisi zone and Wondogenet district. A: East-African-Indian (L3); B: Euro-American lineage (L4); C: Lineage 7 (Ethiopian lineage).

**Figure 5.**
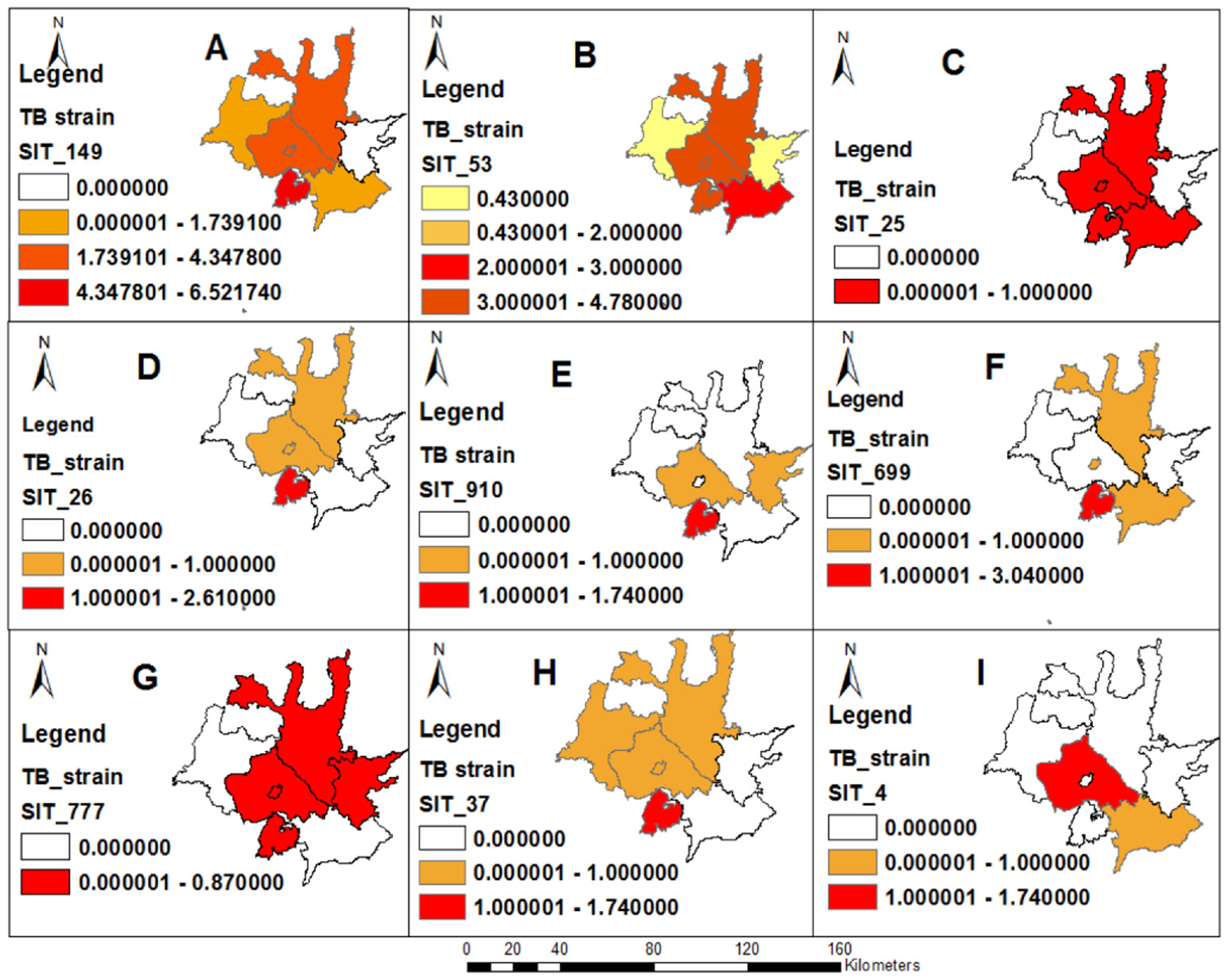
Spatial distribution of clustered strains by district; West Arisi zone and Wondogenet district. SIT: Spoligotype international type. A: SIT 149; B: SIT 53; C:SIT 25; D:SIT 26; E: SIT 910; F:SIT 699; G:SIT 777; H: SIT 37; I: SIT 4.

The spatial distribution of lineages identified in the study is presented in Figure 4. Mapping of the geographic location of clustered strains using GPS showed that the distribution of clustered strains varies within districtsand the highest proportions of clustered strains were observed in Wendogenet district of Sidama Region with SIT 149 (6.5%), SIT 699 (3.0%)and SIT 25 (2.6%). Shashemene town also had a higher proportion of clustered strains such as SIT 149 (4.4%), SIT 53 (4.8%) than other districts. The distribution of clustered strains varied across districts. Wondogenet had all types of TB clusters. (Figure 5).

### Drug susceptibility profile

Drug susceptibility testing was carried out on 202 of 230 *M. tuberculosis* isolates for the first-line drugs: INH, RPM, EMB and for STM. A total of 29 (14.3%) isolates were resistant to any of the drugs tested. The highest monoresistance was observed for INH in 10.9% (22). No MDR-TB was detected in the current study (Table 2).

### Genotyping of drug resistant strains

Genotyping of the drug-resistant strains showed SIT 149 (T3-ETH) to be dominant strain (9/43) among the drug resistant isolates followed by SIT 53 (3/43) and SIT 390 (2/43).

## Discussion

The study revealed a heterogeneous pool of *M. tuberculosis* strains with several clusters including lineage 7 strains circulating in south Ethiopia. A high proportion of INH resistance was reported in the current study and SIT 149 (T3-ETH) was the most dominant circulating strain in the study area including among drug-resistant cases. The high clustering of strains by geographic location suggest the ongoing transmission of TB, including of drug-resistant TB in southern Ethiopia and calls for surveillance and wider monitoring of DST and improved control responses.

In the current study, the majority of the isolates (82%) belonged to the Euro-American lineage (L4) followed by East-Africa-Indian (L3), 14% and the Ethiopian lineage (L7), 4%. A recent study in southern Ethiopia (which was geographically close to our study) reported that 84% of the isolates were L4 [20]. Studies from other parts of the country reported variable proportion of lineage types in different geographic areas of Ethiopia [9, 21]. Overall, L4 is more predominant than all other lineages combined in Ethiopia [2,12] which is in line with our finding. Compared to other African countries, L4 was also predominantly found in Eritrea, Kenya and Uganda [2,22] while a higher proportion of L3 (25–35%) was reported from northern Ethiopia than elsewhere [9]. From African countries, L3 was predominantly reported from Sudan [2] which is geographically close to northern Ethiopia. Our finding showed lower prevalence of L3 in southern Ethiopia compared to northern Ethiopia.

Lineage 7 (Ethiopian lineage) accounted for 4% in the current study. One case of Lineage 7 was recently reported from the southern part of Ethiopia [20] and 6 cases (2%) from the southwest [23]. Lineage 7 was first reported from Woldia in Amhara region of Ethiopia with a prevalence rate 13% [9] and later 16% in the same region [24]. So far, lineage 7 has been prominently reported from the northern part of Ethiopia. The additional report of lineage 7 in the current study suggests its broader occurrence, including in the southern parts of the country. Considering the pre-modern split of this lineage in the phylogenetic tree of *M. tuberculosis* and its localization to Ethiopia only, further investigations into its epidemiology would be of much interest. This study indicated slight difference in the genetic diversity of *M. tuberculosis* in the southern Ethiopia compared to other parts of the country. The wider implication of this on the dynamics of the transmission of TB and drug resistance in the area has yet to be investigated well.

In the current study, SIT 149 was the most common spoligotype (21%) circulating in the study area. Previous studies in Ethiopia have indicated that SIT149, also known as T3-ETH (29), ETH-3 and more recently as L4.2.ETH1 [2], is the most common spoligotype widely distributed in the country [12]. It is also known to be more frequently associated with drug resistance than other spoligotype clusters [25]. It is important that the distribution of this spoligotype is closely monitored and its drivers identified to better tailor control efforts.

Clustering of strains is a marker for recent transmission [26] and indicates where to target interventions. Mapping of clustered strains using GIS has particular importance as it helps to locate cluster position of strains and thus describes the epidemiological links of *M. tuberculosis* strains to a specific geographic locality [12]. In this study, GPS mapping demonstrated the presence of *M. tuberculosis* strain clusters in different districts and identifying areas affected with possible recent transmission. To our knowledge, this is the first report in the country on mapping of *M. tuberculosis* clustered strains position in the community. Although spoligotyping may correctly identify *M. tuberculosis* complex in to various lineages and sub-lineages [7], it is known to overestimate clustering of isolates [27]. In our study, Shashemene town and Wendogenet district are areas with high proportion of clustered strains and this call for the need of strengthening TB control activities in the areas to curb the transmission of the disease.

In the current study, 14% of the newly diagnosed TB patients were resistant to ≥1 first-line anti-TB drugs which is lower than reports from other parts of the country, as high as 23% in Central [11] and 23% in Eastern [5], but relatively higher than the prevalence rate of 11% reported in Northern Ethiopia [4] and 9 % in Southern Ethiopia [28]. The difference in the prevalence rates observed in different parts of the country could be due to differences in TB control program performance, population dynamics, methods or study periods. It is lower than reports from Nairobi which is as high as 30% [29].

INH monoresistance was 10.9% in the current study and it is comparable with reports from eastern Ethiopia, 9.5% [5], but lower than 13.2% from western [30] and higher than reports from central Ethiopia, 4.7% [11]. No MDR-TB was reported in the current study. However, in terms of ordering of drug resistance acquisition, study from South Africa [31] showed that isoniazid resistance was the initial resistance mutation to be acquired in drug resistant TB and is the common pathway for the development of MDR-TB. Therefore, the relatively high INH monoresistance that was observed in the current study should alert to the potential development of MDR TB in the study area and highlights the need for program based DST monitoring.

Linking strain typing data with data on drug resistance can be a useful way to monitor the spread of individual drug-resistant clones in communities [6].T3-ETH (SIT 149) was the most prevalent spoligotype (21%) among drug resistant strains in this series. Fifty percent (12/24) of the drug resistant *M. tuberculosis* isolates were SIT 149 in a collection dating from 2006–2010 [25] and T3-ETH (SIT 149) was associated with MDR-TB [10]. T3-ETH (SIT 149) is the predominant spoligotype cluster associated with drug resistance in Ethiopia [24]. However, the observed association between T3-ETH (SIT 149) and development of drug resistance may not necessarily indicate that these strains are more prone to be drug-resistant but could rather be a consequence of their high prevalence in the population [25]. The correlation between genotypes and TB drug resistance was still uncertain [32]. Further analysis on SIT149 identified genotype SIT149:A, a potential MDR-TB clone circulating in the Ethiopian highlands probably contributes to the spread of MDR-TB in the area that warrants further attention [25].

### Limitations

The study had certain limitations. First, as our study participants were only newly diagnosed pulmonary TB cases, it was not possible to assess the magnitude of drug resistant TB in the previously treated TB and in extrapulmonary TB cases and the strain types between these groups. This has limited us from assessing the overall burden of drug resistant TB in the study area. Second, study participants were all patients seeking treatment at health facilities. Findings from such a selected population may overestimate the true burden of the problem at community level. Third, though Spoligotyping is a robust tool, in mixed TB infection; it is difficult to differentiate between *M.tuberculosis* strains; therefore, we did not asses mixed TB infection in our study.

## Conclusions

The study identified heterogeneous pool of *M. tuberculosis* in different clusters, and high proportion of INH monoresistance. SIT 149 (T3-ETH) was the most dominant strain cluster circulating in the study area, including among drug resistant cases. The study highlights an ongoing transmission of TB, including of drug resistant TB, in southern Ethiopia and call for surveillance and wider monitoring of DST, supported by geospatial analysis to monitor transmission trends and improve control responses.

## Figures and Tables

**Table 1.** Sociodemographic characteristics and clinical variables of study participants, Southern Ethiopia (N = 250).

Characteristics	N (%)
**Sex**	
Male	145 (58.0)
Female	105 (42.0)
**Age in years**	
< 14	14 (5.6)
15–34	193 (77.2)
35–44	23 (9.2)
45–54	9 (3.6)
≥ 55	11 (4.4)
**Location**	
Urban	107 (42.8)
Rural	143 (57.2)
**Marital status**	
Single	109 (43.6)
Married	129 (51.6)
Other	12 (4.8)
**Education**	
Primary school and above	195 (78.0)
Illiterate	55 (22.0)
**Occupation**	
Farmer	79 (31.6)
Student	57 (22.8)
Housewife	38 (15.2)
Government employee	9 (3.6)
Other	67 (26.8)
**HIV serostatus**	
Positive	10 (4.0)
Negative	236 (96.0)
Not tested	4 (1.6)

**Table 2.** Resistance to first-line anti-TB drugs and to streptomycin among sputum positive TB in Southern Ethiopia.

Total tested	Number (n = 202)	Percentage (%)	95% CI
Pan sensitive	173	85.7	80.03 – 90.16
Any resistance	29	14.3	9.83 – 19.96
**Monoresistance**			
INH	22	10.9	6.92 – 16.02
RPM	1	0.5	0.01 – 2.72
STM	1	0.5	0.01 – 2.72
EMB	5	2.5	0.80 – 5.68
**Combined drug resistance**			
INH+RPM	0	0	
INH + EMB	1	0.5	0.01 – 2.72
INH + STM	3	1.4	0.30 – 4.27
INH+RPM+STM+EMB	0	0	

INH: Isoniazid; EMB: Ethambutol; RPM: Rifampicin; STM: Streptomycin.
